# Forecasting Spoken Language Development in Children With Cochlear Implants Using Preimplant Magnetic Resonance Imaging

**DOI:** 10.1001/jamaoto.2025.4694

**Published:** 2025-12-26

**Authors:** Yanlin Wang, Di Yuan, Shani Dettman, Dawn Choo, Emily Shimeng Xu, Denise Thomas, Maura E. Ryan, Patrick C. M. Wong, Nancy M. Young

**Affiliations:** 1 Brain and Mind Institute, The Chinese University of Hong Kong, Hong Kong Special Administrative Region (SAR), China; 2 Department of Psychology, The Chinese University of Hong Kong, Hong Kong SAR, China; 3 Department of Audiology and Speech Pathology, The University of Melbourne, Parkville, Victoria, Australia; 4 Division of Otolaryngology Head & Neck Surgery, Ann & Robert H. Lurie Children’s Hospital of Chicago, Chicago, Illinois; 5 Department of Audiology, Ann & Robert H. Lurie Children’s Hospital of Chicago, Chicago, Illinois; 6 Department of Medical Imaging, Ann & Robert H. Lurie Children’s Hospital of Chicago, Chicago, Illinois; 7 Department of Linguistics and Modern Languages, The Chinese University of Hong Kong, Hong Kong SAR, China; 8 Department of Otolaryngology–Head and Neck Surgery, Feinberg School of Medicine, Northwestern University, Chicago, Illinois; 9 Knowles Hearing Center, Department of Communication Sciences and Disorders, Northwestern University, Evanston, Illinois

## Abstract

**Importance:**

Cochlear implants substantially improve spoken language in children with severe to profound sensorineural hearing loss, yet outcomes remain more variable than in children with healthy hearing. This variability cannot be reliably predicted for individual children using age at implant or residual hearing. Development of an artificial intelligence clinical tool to predict which patients will exhibit poorer improvements in language skills may enable an individualized approach to improve language outcomes.

**Objective:**

To compare the accuracy of traditional machine learning (ML) with deep transfer learning (DTL) algorithms to predict post–cochlear implant spoken language development in children with bilateral sensorineural hearing loss using a binary classification model of high vs low language improvers.

**Design, Setting, and Participants:**

This multicenter diagnostic study enrolled children from English-, Spanish-, and Cantonese-speaking families across 3 independent clinical centers in the US, Australia, and Hong Kong. A total of 278 children with cochlear implants were enrolled from July 2009 to March 2022 with 1 to 3 years of post–cochlear implant outcomes data. All children underwent pre–cochlear implant 3-dimensional volumetric brain magnetic resonance imaging (MRI). ML and DTL algorithms were trained to predict high vs low language improvers in children with cochlear implants using neuroanatomical features from presurgical brain MRI. Data were analyzed from August 2023 to April 2025.

**Exposures:**

Cochlear implants.

**Main Outcomes and Measures:**

The accuracy, sensitivity, and specificity of prediction models based on brain neuroanatomic features using traditional ML and DTL learning.

**Results:**

Of 278 children, 137 (49.3%) were female, and the mean (SD) age at cochlear implant was 25.7 (18.8) months. DTL prediction models using bilinear attention-based fusion strategy achieved an accuracy of 92.39% (95% CI, 90.70%-94.07%), sensitivity of 91.22% (95% CI, 89.98%-92.47%), specificity of 93.56% (95% CI, 90.91%-96.21%), and area under the curve of 0.98 (95% CI, 0.97-0.99). DTL outperformed traditional ML models in all outcome measures.

**Conclusions and Relevance:**

The results of this diagnostic study suggest that DTL prediction of language improvement on the individual child level using neuroanatomic features demonstrates greater accuracy, sensitivity, and specificity than traditional ML prediction. DTL was substantially improved by direct capture of discriminative and task-specific information that are advantages of representation learning enabled by this approach vs ML. The results support the feasibility of a single DTL prediction model for language prediction for children served by cochlear implant programs worldwide. Prediction of low improvement may enable targeted early and customized intervention to improve language.

## Key Points

**Question:**

Can deep transfer learning (DTL) achieve superior accuracy, sensitivity, and specificity vs traditional machine learning when predicting cochlear implant improvements across heterogeneous, multicontinental datasets from diverse populations?

**Findings:**

In this multicenter cohort study of 278 pediatric cochlear implant recipients spanning 3 languages (English, Spanish, and Cantonese), a bilinear attention-based DTL model achieved 92.39% accuracy in forecasting spoken language improvement, demonstrating superior sensitivity and specificity vs conventional approaches.

**Meaning:**

The findings of this study underscore the scalability and robustness of DTL applied to preimplant magnetic resonance images with heterogeneous data, potentially offering a prognostic tool for forecasting spoken language development in children with cochlear implants.

## Teaser

This diagnostic study examines the accuracy of machine learning with deep transfer learning algorithms to predict post–cochlear implant spoken language development in children with bilateral sensorineural hearing loss.

## Introduction

Cochlear implants are an effective treatment for young children with severe to profound sensorineural hearing loss (SNHL) that enables development of spoken language.^1^ However, language outcomes after cochlear implant are variable compared with children with healthy hearing.^2^ Despite the availability of various early intervention approaches, there is little consensus on the optimal type and dose of behavioral therapy to improve listening and spoken language.^3^ Accurate prediction of spoken language development on the individual child level before cochlear implant may allow for a customized predict to prescribe approach to reduce outcome variability.^4^ Accurate prediction of spoken language improvement, in particular for parents of children likely to achieve lower language improvement, has the potential to improve pre–cochlear implant parental counselling and post–cochlear implant therapy planning. Prediction gives parents and therapists the opportunity to arrange for more intensive behavioral therapy. Moreover, by forecasting the language developmental trajectory after cochlear implant, it becomes possible to evaluate the efficacy of different therapy approaches. Neural prediction may lead to development of more effective therapies based on pre–cochlear implant brain structure and function.

Brain measures serve as better prognostic indicators, either alone or in combination with other measures, than traditional measures, such as age at implant and preimplant residual hearing.^5^ Early auditory experience is associated with the development of auditory and language networks that are crucial for subsequent growth.^7^ Studies have successfully used machine learning (ML) techniques to forecast the auditory and spoken language skills of children with cochlear implant. For example, using the presurgical neuroanatomical features from magnetic resonance imaging (MRI) and an ML linear support vector machine classifier, a prediction accuracy of 84% was achieved as to whether a child would experience high vs low speech perception improvement 6 months after device activation, as assigned by the group median outcome score.^11^ In comparison, nonneural features, including age at implant and residual hearing, only reached a chance level of accuracy in predicting speech perception improvement. The robustness and efficiency of brain measures in predicting post–cochlear implant improvements in children and adults have also been supported by studies that used brain imaging techniques to activate brain regions with audio and visual stimuli.^12^

Despite the increasing number of studies that use ML to predict post–cochlear implant outcomes, modeling brain data on a multicenter dataset remains challenging due to the variations in MRI scan protocol and outcome measurements.^15^ Another complicating factor is that applying a dimensional reduction (an essential preprocessing step for many ML algorithms) to heterogeneous datasets can oversimplify the data, leading to overfitting, reduced interpretability, and ultimately diminished model effectiveness.^16^ Deep learning has shown considerable advantages in representation learning, the ability to automatically learn from useful data sources, and scalability vs ML in modeling brain imaging data.^16^ Deep transfer learning (DTL) can leverage prior knowledge learned from pretraining on a large dataset to enhance downstream task performance.^18^ Multimodal integration can identify feature value patterns across modalities (eg, clinical findings, such as age at implant and residual hearing; brain neuroanatomy imaging findings), which is essential, as it affects how well a model learns and generalizes across multiple data sources.^20^ However, the variability in feature distributions and outcome measures across centers and languages fundamentally challenges the capability of deep learning to identify how information is encoded and processed by the brain, referred to as *discriminative brain representations*, for predicting cochlear implant improvement on a multicenter dataset.^22^ Consequently, rigorous evaluation of the robustness of deep learning approaches on the multicenter dataset is imperative before using deep learning–based preoperative neural prediction models.

To this end, this study developed and compared neural predictive models for forecasting long-term post–cochlear implant improvements in children with cochlear implants and evaluated their performance and robustness on the multicenter dataset. The working hypothesis of the study was that neural prediction modeling done with DTL would be more accurate, sensitive, and specific than traditional ML when applied to a heterogenous dataset from cochlear implant programs located in different continents serving diverse populations.

## Methods

### Participants

Children with congenital or early-onset SNHL who received cochlear implants between July 2009 and March 2022 were enrolled from 3 international centers: Chicago, Illinois; Melbourne, Australia; and Hong Kong, China. All the children underwent T1-weighted structural whole-brain MRI as a part of their pre–cochlear implant evaluation using each medical center’s standard clinical protocol. Language evaluation was obtained before and after cochlear implant for up to 3 years. This study was approved at each center by the Joint Chinese University of Hong Kong–New Territories East Cluster Clinical Research Ethics Committee, the Stanley Manne Children’s Research Institute’s institutional review board, and The Royal Children’s Hospital human research ethics committee. We obtained written informed consent from all participants and we followed the Strengthening the Reporting of Observational Studies in Epidemiology (STROBE) reporting guideline.

As a study that aimed to predict improvements in as many children with cochlear implants as possible, we imposed relatively broad inclusion and exclusion criteria. Children had to be from homes in which the dominant family language spoken was Cantonese (Hong Kong), English (Melbourne, Chicago), or Spanish (Chicago). Children with additional conditions known to be associated with language development (eg, Down syndrome, Fragile X, and autism spectrum disorder) independent of SNHL were excluded, as were children with gross brain malformations. In addition, correlation matrix analyses of demographic variables were performed for each center (eMethods in Supplement 1).

### Clinical Measures

Children’s auditory skill, speech perception, and receptive and/or expressive language abilities were measured before and up to 36 months after implant using different assessment tools across centers (eMethods in Supplement 1). We referred to all these measurements as spoken language, being aware that audition and speech perception are precursors for spoken language development.^23^ Positive correlations have been demonstrated between speech perception and spoken language scores on standardized tests for children with hearing loss.^25^ While variances could be introduced by differences in the assessment methods and timing, it is feasible to compare the spoken language ability across the centers and over time because of the heterotypic stability inherent in spoken language development.^27^ Specifically, a child’s spoken language rank order in the population remains consistent across age as long as those characteristics share the same underlying construct and theoretical value. Therefore, instead of using the raw scores directly for fine-grained prediction, we separated the spoken language improvement into binary classifications (high improvement and low improvement) using a median split approach for children within each center.

The improvement of spoken language development from pre–cochlear implant to post–cochlear implant was quantified by the change of assessed scores as a function of assessment time for each participant. To this end, a linear mixed-effect model was constructed for each center with spoken language scores as the dependent variable, participant identification (ID) as a random intercept, and assessment time as a random slope. The fixed-effects portion of the model included only the intercept term, as the association of time with spoken language scores was captured in the random slope. The model can be expressed mathematically as scores − 1 + (assessment time | participant ID). The random slope in the model allowed us to estimate individual differences in the rate of speech and language change over time. Children with slope values larger than the group median were labeled as having high improvement, while those with slope values smaller than the group median were labeled as having low improvement.

### MRI Acquisition and Preprocessing

The T1-weighted MRI image was obtained from each child before cochlear implant. The scanning parameters were optimized to obtain a good signal-to-noise ratio (eMethods in Supplement 1). MRI images were processed using the advanced normalization tools in Python, version 3.8.19.^31^ To increase the image quality, the images were resampled to 1 × 1 × 1 mm voxel size and preprocessed following the basic preprocessing pipeline for T1-weighted brain MRI in advanced normalization tools. The deformation-based morphometry method was used to examine the morphological differences over the entire brain with an age appropriate T1 image as the template.^32^ Fifteen axial 2-dimensional slices were extracted from the central part of the 3-dimensional deformation-based morphometry brain scans.^34^ The images were cropped and resized into a target resolution of 128 × 128 voxels and normalized using ImageNet statistics (mean = [0.485, 0.456, 0.406], standardized difference = [0.229, 0.224, 0.225]) before being passed on for further analyses.^35^ Each slice was assigned the same label as the corresponding participant and used as a data sample to train the model. In addition, we conducted a sensitivity analysis to assess potential bias arising from slice selection, evaluating model performance across different slice counts or positions. The detailed results of this analysis are provided in the eMethods in Supplement 1.

### Transfer Learning and Feature Extractions

Pretrained convolutional neural network (CNN) models used included AlexNet,^36^ VGG19,^37^ ResNet,^38^ GoogleNet,^39^ Inception,^40^ MobileNet,^41^ and DenseNet,^42^ as implemented in PyTorch, version 1.9, for feature extraction. This standard transfer learning strategy involves using pretrained CNN models on ImageNet as the backbone of the model to capture generalizable features, followed by fine-tuning the top layers to learn new specialized representations that were tailored to our output classifier.^36^ During the fine-tuning phase, the weights and biases of the CNN models were frozen to prevent changes. Subsequently, an attention-based fusion network was added to incorporate clinical measures into neural feature representations from the hidden layer’s activation function to achieve a higher performance of the model by using a bilinear attention mechanism. Specifically, clinical measures included age at cochlear implant, age at MRI, age at hearing aid fitting, sex, left/right pure tone average residual hearing, and preoperative language ability scores. We used a bilinear attention network to capture high-level interactive relations among multiple modalities and then extracted joint image meta representation by a bilinear pooling layer.^44^ Data augmentation with random rotation and flipping was executed to improve the model training efficiency.^46^ The loss function was binary cross-entropy with logit loss. The optimizer was Adam with a learning rate of 1 × 10^-4^. A total of 100 epochs with a batch size of 64 images were set for training. The validation performance was used to determine when to stop the training. The CNN models were trained until there was no improvement in the validation loss for 10 consecutive epochs. The model’s performance was validated using 5-fold cross-validation on 80% of the data, with the remaining 20% used as a held-out test set for evaluation.

### Performance Comparisons

To examine whether neural features can predict long-term post–cochlear implant improvements, we first compared state of the art CNN models in the multicenter dataset. To evaluate whether our model’s performance was robust to variations in data distribution across medical centers and languages, we evaluated the model performance on single datasets or combined datasets. Moreover, to improve the performance of the neural predictive model, we integrated clinical features with neural features by using a bilinear attention mechanism. In addition, to compare the effectiveness of DTL and traditional ML models in capturing shared and robust brain representations, we evaluated both approaches on a prediction task using a multicenter dataset. Seven DTL models and 8 ML models were compared: Lasso regression, Ridge regression (RR), support vector machine, random forest, decision tree, K-nearest neighbor, and eXtreme Gradient Boosting (XGBoost). To reduce the dimensionality of the whole-brain voxel-wise features, we applied 4 linear and nonlinear dimensionality reduction techniques^16^: principal component analysis, Gaussian random projection, recursive feature elimination, and univariate feature selection (UFS). The detailed information on ML models and dimensionality reduction methods is provided in the eTable in Supplement 1.

## Results

**Figure 1.**
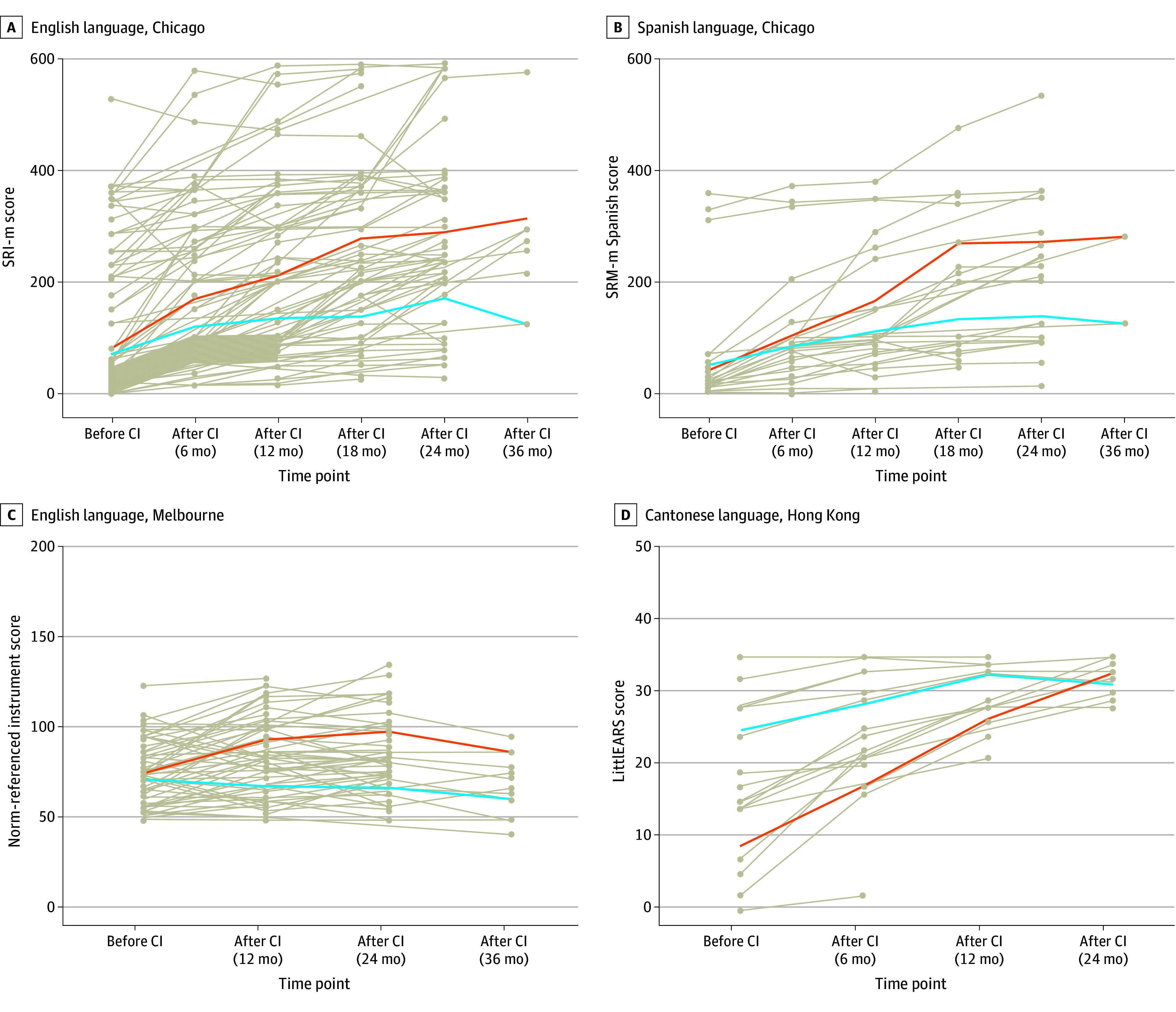
Spoken Language Ability of Children Before and After Cochlear Implant (CI) at Each Center A, In the Chicago English group, the spoken language ability was measured using Recognition Index–modified version (SRI-m), a hierarchical battery ranged from 0 to 600. B, In the Chicago Spanish group, the spoken language ability was measured using SRI-m Spanish version. C, In the Melbourne English group, the spoken language ability was assessed using 2 norm-referenced instruments: the Picture Peabody Vocabulary Test–4 and Preschool Language Scale 4 and 5. Standard scores were 100 (±15 was the normative mean). D, In the Hong Kong Cantonese group, the LittlEARS Auditory questionnaire was used, with the score ranging from 0 to 35. Each gray line represents the spoken language development trajectory for 1 child. The orange line represents the mean spoken language score for the high-improvement group, and the blue line represents the mean spoken language score for the low-improvement group.

A total of 278 children were included. The demographic information is shown in Table 1. Children with cochlear implants showed improvements in spoken language abilities compared with the baseline measurement tested before implant (Figure 1). Specifically in Chicago, the mean (SD) spoken language abilities of English-learning children improved from 75 (114) to 292 (129) and those of Spanish-learning children from 45 (90) to 203 (110) during the period from pre–cochlear implant to 36 months post–cochlear implant, as tested by SRI-m. Similarly, in Hong Kong, Cantonese-learning children showed an increase in mean (SD) scores from 17 (10) to 32 (3) during the period from pre–cochlear implant to 24 months post–cochlear implant as measured by the LittlEARS Auditory Questionnaire (range, 0-35). The rate of improvement was greatest during the first 1.5 years after initial implant. In Melbourne, the mean (SD) receptive language of English-learning children improved from 74 (16) to 85 (21) during the first 2 years after implant but dropped to 70 (16) during the third year post–cochlear implant as tested by the Picture Peabody Vocabulary Test–4 and Preschool Language Scale 4 and 5 (standard score, mean [SD]: 100 [15]) (Table 1 and eFigure in Supplement 1). The different pattern of changes in spoken language development may have resulted from the standard scores obtained in Melbourne, which took age-appropriate children with healthy hearing as a control, suggesting that children were able to catch up with their peers with healthy hearing but still lagged behind in their long-term spoken language development. Despite different standardized tests being used to capture the spoken language development across the centers, our predictive models were constructed to only predict the binary classifications of low or high improvement.

**Table 1.** Demographic Information for Participants From Different Centers

Characteristic	No. (%)				All
	Chicago	Chicago	Melbourne	Hong Kong	
Sample size, No.	143	37	81	17	278
Family language spoken	English	Spanish	English	Cantonese	NA
Female	67 (46.9)	21 (56.8)	37 (45.7)	12 (70.6)	137 (49.3)
Male	76 (53.1)	16 (43.2)	44 (54.3)	5 (29.4)	141 (50.7)
Age at SNHL diagnosis, mean (SD), mo	10.2 (13.3)	11.1 (12.4)	3.2 (4.4)	11.6 (15.2)	9.7 (12.8)
Age at HA fitting, mean (SD), mo	11.6 (13.2)	12.3 (12.5)	3.8 (4.2)	16.9 (13.6)	10.4 (12.3)
Age at MRI, mean (SD), mo	23.8 (20.5)	26.9 (18.2)	11.4 (12.1)	24.3 (18.0)	20.7 (18.9)
Age at CI, mean (SD), mo	27.4 (20.9)	30.1 (18.4)	19.2 (13.2)	32.5 (16.6)	25.7 (18.8)
Unaided hearing of left ear, mean (SD), dB HL	95.4 (17.0)	98.9 (18.0)	97.7 (18.7)	103.3 (15.7)	96.9 (17.5)
Unaided hearing of right ear, mean (SD), dB HL	93.7 (18.1)	100.2 (15.1)	99.5 (19.0)	101.7 (14.0)	96.5 (17.9)
SES, mean (SD)^a^	86 050.17 (31 011.63)	63 300.58 (14 334.92)	1352 (679.98)	Unknown	NA
Device manufacturer (sample size)	Cochlear Americas (71); Advanced Bionics (24); Med-EI (48)	Cochlear Americas (24); Advanced Bionics (6); Med-EI (7)	Cochlear Americas	Cochlear Americas	NA
Device configuration (sample size)	CI: sequential (53); CI: simultaneous (51); CI: unilateral (10); bimodel (29)	CI: sequential (21); CI: simultaneous (3); CI: unilateral (8); bimodel (5)	CI: sequential (22); CI: simultaneous (36); unknown (23)	CI: simultaneous (7); CI: unilateral (1); bimodel (1); unknown (8)	NA
**All**					
Pre-CI, mean (SD)	75.09 (114.23)	45.58 (89.58)	74.21 (16.28)	16.81 (10.44)	NA
Post-CI, mean (SD)					
6 mo	145.07 (118.51)	93.76 (90.09)	Not tested	22.83 (8.91)	NA
12 mo	177.34 (134.16)	136.41 (106.98)	81.30 (20.29)	29.00 (4.20)	NA
18 mo	223.19 (139.10)	192.76 (126.44)	Not tested	NA	NA
24 mo	249.17 (132.94)	201.70 (134.79)	84.68 (21.37)	32.00 (2.73)	NA
36 mo	291.50 (128.87)	203.13 (110.49)	69.6 (16.29)	NA	NA
**Low-improvement group**					
Pre-CI, mean (SD)	71.76 (119.41)	47.66 (101.77)	72.12 (15.77)	24.75 (7.25)	NA
Post-CI, mean (SD)					
6 mo	119.75 (108.41)	84.68 (98.43)	Not tested	28.50 (5.61)	NA
12 mo	133.48 (114.73)	110.46 (109.28)	68.66 (13.85)	32.6 (2.70)	NA
18 mo	136.24 (99.89)	132.31 (115.13)	Not tested	Not tested	NA
24 mo	169.85 (134.27)	137.60 (120.02)	76.68 (10.93)	31.25 (3.30)	NA
36 mo	125 (NA)	125 (NA)^b^	62 (12.22)	Not tested	NA
**High-improvement group**					
Pre-CI, mean (SD)	78.51 (109.40)	43.26 (76.76)	76.14 (16.72)	8.88 (6.10)	NA
Post-CI, mean (SD)					
6 mo	170.38 (123.65)	103.98 (81.64)	Not tested	17.17 (8.13)	NA
12 mo	212.57 (139.04)	164.52 (101.41)	93.95 (17.73)	26.43 (2.99)	NA
18 mo	276.94 (133.15)	268.31 (100.23)	Not tested	Not tested	NA
24 mo	288.83 (114.08)	272.92 (117.60)	98.04 (17.75)	32.75 (2.22)	NA
36 mo	315.29 (118.72)	281.25 (NA)^a^	87.33 (8.50)	Not tested	NA

Abbreviations: CI, cochlear implant; HA, hearing aid; MRI, magnetic resonance imaging; NA, not applicable or not available; SES, socioeconomic status; SNHL, sensorineural loss.

^a^ SES was measured by household income (US$) in the Chicago sample and by the Socio-Economic Indexes for Areas in the Melbourne sample.

^b^ Only 1 set of data was available, and the SD could not be calculated.

**Figure 2.**
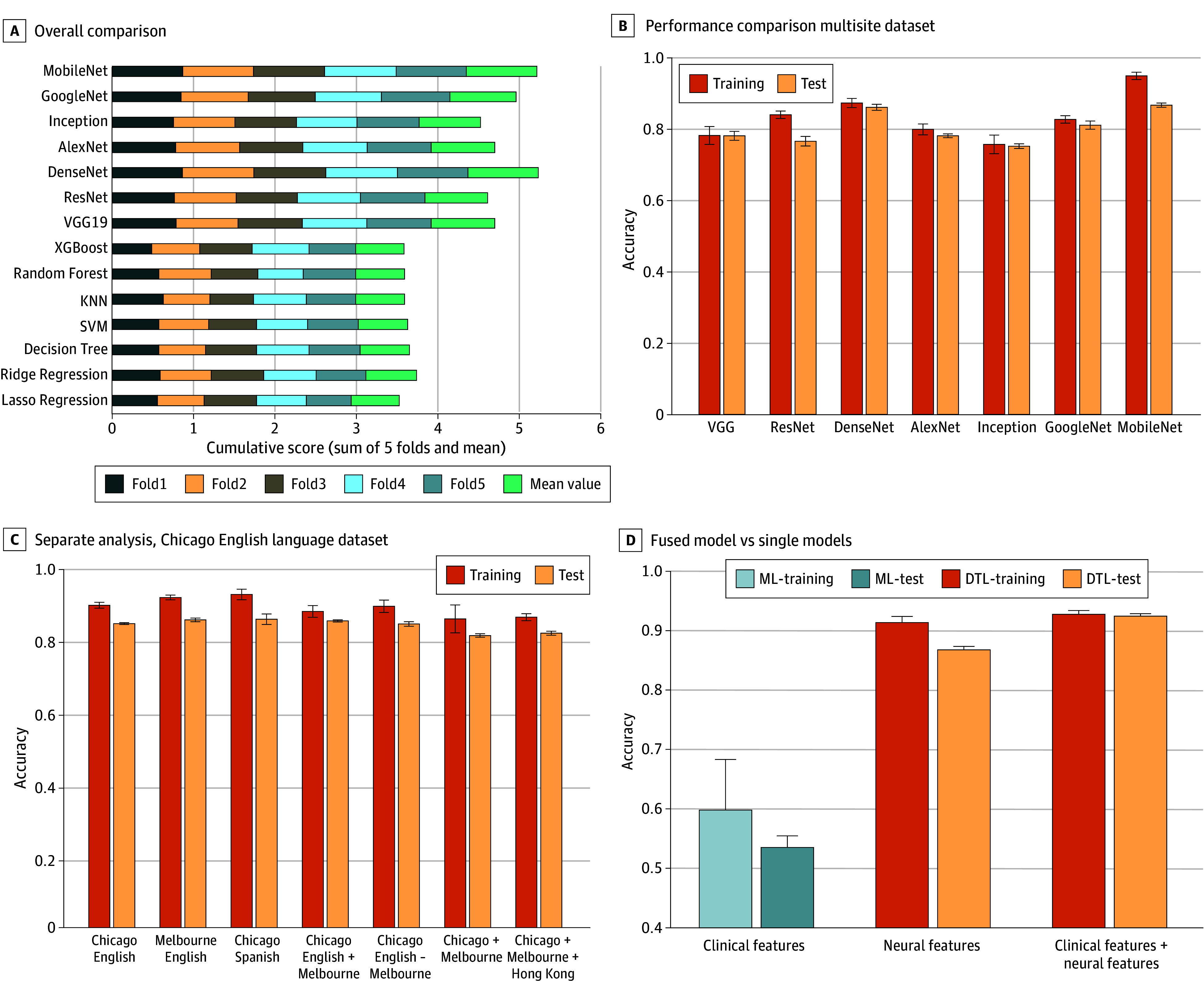
Exhaustive Evaluation of Model Performance Comparison Across Datasets and Modalities A, Overall comparison of machine learning models vs deep transfer learning models on the multisite dataset. B, Performance comparison among different transfer learning models on the multisite dataset. C, Evaluation of the deep transfer learning model separately on the Chicago-English dataset, across languages or the cohort centers, and on the combined dataset. D, Performance comparison between the fused model and single models. Whiskers in C and D indicate confidence intervals determined from 5-fold cross-validation of the training data and the independent test set.

Table 2 lists the training and testing of accuracy, sensitivities, specificities, and area under the curve (AUC) for the DTL and standard ML models. The results showed that DTL models can substantially improve the model’s prediction performance compared with ML models with the UFS dimensionality reduction method (Figure 2A). Among the various deep learning CNN models, the MobileNet model exhibits the best performance, with an accuracy of 86.79% (95% CI, 85.398%-87.60%) and AUC of 0.924 (95% CI, 0.92-0.93) on the test dataset. In contrast, Ridge with UFS exhibited the superior performance, with an accuracy of 62.14% (95% CI, 59.25%-65.03%) and AUC of 0.621 (95% CI, 0.59-0.65) compared with the other 3 dimensionality reduction approaches (eTable in Supplement 1). This indicated that DTL models can learn general and domain-specific feature representations through the pretrained and finetuning procedure, yielding higher performance than standard ML models trained on lower-dimensional projections of high-dimensional inputs.

**Table 2.** Performance Comparison of Deep Transfer Learning and Machine Learning Models on the Multicenter Dataset

Types/models	% (95% CI)			AUC (95% CI)
	Accuracy	Sensitivity	Specificity	
Slice based				
VGG19-bn	78.15 (76.41-79.90)	76.00 (74.38-77.62)	80.09 (77.84-82.34)	0.867 (0.78-0.82)
ResNet-50d	76.68 (74.84-78.52)	76.63 (72.71-80.55)	76.60 (70.48-82.72)	0.86 (0.84-0.87)
DenseNet-169	86.14 (85.80-86.48)	85.22 (84.43-86.00)	86.97 (86.05-87.90)	0.90 (0.90-0.91)
AlexNet	78.15 (77.41-78.89)	82.55 (79.90-85.20)	75.59 (73.36-77.82)	0.84 (0.83-0.85)
Inceptio-V3	75.24 (74.34-76.14)	77.19 (71.96-82.42)	73.12 (66.60-79.64)	0.83 (0.82-0.84)
GoogleNet	81.10 (79.81-82.40)	81.52 (79.65-83.39)	80.73 (79.02-82.43)	0.87 (0.87-0.87)
MobileNet	86.79 (85.98-87.60)	89.90 (88.18-91.63)	83.74 (81.24-86.25)	0.92 (0.92-0.93)
Voxel based^a^				
Lasso	58.57 (53.76-63.38)	51.43 (44.01-58.85)	65.71 (61.75-69.68)	0.59 (0.54-0.63)
Ridge	62.14 (59.25-65.03)	55.72 (47.07-64.36)	68.57 (61.28-75.59)	0.62 (0.59-0.65)
DT	60.71 (56.57-64.86)	42.86 (31.99-53.72)	78.57 (64.20-92.94)	0.61 (0.57-0.65)
SVM	60.36 (57.47-63.25)	55.71 (53.29-58.14)	65.00 (59.22-70.78)	0.60 (0.58-0.63)
KNN	59.64 (54.30-64.98)	56.43 (45.84-67.02)	62.86 (54.80-70.91)	0.60 (0.54-0.65)
RF	59.64 (54.30-64.98)	47.86 (38.66-57.05)	71.43 (68.29-74.56)	0.60 (0.54-0.65)
XGBoost	59.64 (49.65-69.63)	55.00 (42.22-67.78)	64.29 (51.36-77.21)	0.60 (0.50-0.70)

Abbreviations: AUC, area under the curve; DT, decisions tree; KNN, K nearest neighbor; RF, random forest; SVM, support vector machine; XGBoost, eXtreme gradient boosting.

^a^ Univariate feature selection feature extraction.

Moreover, regardless of whether a single dataset or a combination of different datasets was used to build the model, the MobileNet model demonstrated consistently accurate performance (Figure 2C). Specifically, it achieved an accuracy of 90.36% (95% CI, 89.96%-90.76%) and AUC of 0.947 (95% CI, 0.94-0.95) on the Chicago English-speaking and Melbourne English-speaking datasets (same language, 2 centers) and an accuracy of 89.48% (95% CI, 88.57%-90.39%) and AUC of 0.937 (95% CI, 0.934-0.939) on the Chicago English-speaking and Chicago Spanish-speaking datasets (same center, 2 languages). When tested on the Chicago, Melbourne, and Hong Kong datasets, it achieved an accuracy of 86.79% (95% CI, 85.98%-87.60%) and AUC of 0.924 (95% CI, 0.92-0.93).

To evaluate DTL and ML model performance on the type of data or format of data, referred to as modality, single modality features and combined modality features were evaluated. Compared with the single modality model, the bilinear attention–based fused model achieved the best performance against other single models while the single neural model with MobileNet outperformed the single clinical model with logistic regression (Figure 2D and Table 3). Specifically, the bilinear attention–based fusion model demonstrated superior predictive performance, achieving an accuracy of 92.39% (95% CI, 90.70%-94.07%) and a high AUC of 0.98 (95% CI: 0.97-0.99). This significantly outperformed both baseline models. The model that used only clinical measures showed limited predictive utility, with an accuracy of 53.57% (95% CI, 50.86%-56.29%) and an AUC of 0.52 (95% CI: 0.49-0.56), indicating performance near chance level. The model that used only neural features performed better than the clinical model, with an accuracy of 86.79% (95% CI, 85.98%-87.60%) and an AUC of 0.92 (95% CI: 0.92-0.93), but remained substantially lower than the fusion model.

**Table 3.** Model Performance Comparison Across Datasets and Modalities

Datasets^a^	% (95% CI)			AUC (95% CI)
	Accuracy	Sensitivity	Specificity	
Subsets				
Chicago, English	89.56 (89.18-89.93)	92.02 (90.75-93.29)	86.89 (85.69-88.10)	0.94 (0.94-0.95)
Melbourne, English	90.62 (89.86-91.38)	91.83 (90.30-93.37)	89.43 (88.72-90.14)	0.95 (0.95-0.95)
Chicago, Spanish	90.81 (88.66-92.96)	95.41 (93.71-97.11)	85.20 (81.04-89.36)	0.98 (0.97-0.99)
Chicago, English and Spanish	89.48 (88.57-90.39)	88.64 (88.19-89.04)	90.29 (88.90-91.67)	0.94 (0.93-0.94)
Chicago and Melbourne, English	90.36 (89.96-90.76)	90.65 (88.62-92.69)	90.03 (87.89-92.17)	0.95 (0.94-0.95)
Chicago and Melbourne, English and Spanish	86.00 (85.32-86.68)	81.29 (79.60-82.97)	90.29 (88.85-91.74)	0.90 (0.90-0.91)
All datasets				
Clinical features	53.57 (50.86-56.29)	52.41 (44.18-60.65)	54.81 (47.26-62.37)	0.52 (0.49-0.56)
Neural features	86.79 (85.98-87.60)	89.90 (88.18-91.63)	83.74 (81.24-86.25)	0.92 (0.92-0.93)
Clinical features + neural features	92.39 (90.70-94.07)	91.22 (89.98-92.47)	93.56 (90.91-96.21)	0.98 (0.97-0.99)

Abbreviation: AUC, area under the curve.

^a^ Models for the subsets were constructed using neural features. All datasets comprise Chicago (English and Spanish), Melbourne, and Hong Kong data.

## Discussion

In this multicenter diagnostic study, we used DTL on the preoperative neuroanatomical features obtained from presurgical MRI brain scans to predict up to 3-year spoken language improvements in children with cochlear implants. Our models consistently demonstrated accurate performance in distinguishing between higher and lower improvement groups for a single dataset and combined datasets. A bilinear attention–based fusion model outperformed unimodal approaches by efficiently capturing cross-modal interactions between clinical characteristics and neural imaging features. Our DTL approach demonstrated superior robustness and flexibility in predicting post–cochlear implant improvement from pre–cochlear implant neural data, effectively capturing discriminative and task-specific brain representations across multicenter and language datasets that the current ML methods are not able to match.

To our knowledge, this study represents the largest sample size ever used with brain measures to build a cochlear implant predictive model.^49^ Our evaluation demonstrated that DTL achieves consistently higher accuracies through combined models, confirming its robustness and flexibility to heterogeneous data. These experiments illustrated that DTL can extract the robust, shared feature representations obtained by each medical center from diverse populations. These findings suggest that the inherent heterogeneity arising from factors such as scanner protocols and language outcomes necessitates explicit consideration during model training in multicenter studies to avoid characteristic-specific poor generalization. Furthermore, our results support the concept that preoperative neural features can predict post–cochlear implant improvements in children with diverse backgrounds, regardless of the specific assessment tools used.

The DTL approach has shown to be powerful in health care decisions for rare diseases, such as Alzheimer disease,^52^ cardiomyopathy,^53^ and diabetic retinopathy.^54^ Compared with a previous study by Geng et al^11^ that used voxel-based ML models to predict speech perception improvements 6 months post–cochlear implant with 37 children, our study used a DTL approach and a larger sample size and revealed a higher prediction accuracy, even for long-term post–cochlear implant improvements. Conventional voxel-based ML approaches appear limited in handling heterogeneous multicenter and language data to accurately predict long-term post–cochlear implant improvements. Moreover, dimensionality reduction techniques used to reduce the number of features in a dataset while preserving essential information often fail to extract shared representations from multicenter datasets, fundamentally limiting their effectiveness compared with DTL methods that can exploit such variability.

Deep learning methods demonstrate substantial advantages vs traditional ML approaches in harnessing large, heterogeneous datasets, especially when paired with transfer learning.^55^ For example, Abrol et al^56^ reported that deep learning consistently improved performance at larger training datasets on neuroimaging classification and regression tasks as the sample size increased. While deep learning approaches benefit from larger sample sizes, the inherent heterogeneity of multicenter data necessitates careful handling of representation learning and model development to improve model performance. In our case, transfer learning enabled our models to capture robust, discriminative brain representations, achieving an 87% accuracy for post–cochlear implant outcome prediction on the combined dataset. Furthermore, our novel bilinear attention–based fusion network effectively integrated clinical measures with neural features, substantially enhancing preoperative prediction accuracy to 92.39%. These findings demonstrate that, beyond sample size, effectively leveraging inherent data heterogeneity and multiple modalities is critical for improving model performance and robustness in preoperative neural prediction tasks.

### Limitations

Our study had several limitations. First, the need to accommodate the different outcome measures across centers by using binary classifications (high improvement and low improvement) and a median split method limited the differentiation of children with medium-level outcome measures. Second, processing each 2-dimensional slice independently reduced the spatial information between slices. This was mitigated by using transfer learning and fine-tuning techniques to integrate prior knowledge from large datasets with domain-specific knowledge. Nevertheless, the slice-based approach remains suboptimal for modeling complex volumetric patterns, underscoring the need for future work to incorporate explainable artificial intelligence techniques. Third, the high performance observed in the present study may partially reflect the cohort-specific factors and enlarged number of image inputs inherent to the slice-based approach.^57^ To mitigate this potential overfitting, we used cross-validation and multiple regularization strategies, including dropout, weight decay, and early stopping. Finally, robustness is a prerequisite for deep learning algorithms to generalize across centers.^17^ Although our experiments have demonstrated the robustness of DTL in modeling brain data, cross-center generalization was limited due to variations in features and outcomes across centers. Future research should focus on testing the model’s generalizability across diverse populations and implant programs worldwide.

## Conclusions

This diagnostic study demonstrated the robustness of the DTL approach for neural prediction of whether children will have high or low spoken language improvement after cochlear implant. Furthermore, our model provided more accurate preoperative prediction by using techniques that leverage data from multiple sources to improve performance. This study supports the feasibility of the development of a single accurate DTL neural prediction model to use across centers and languages worldwide. Accurate prediction of spoken language on the individual child level is a first step toward the creation of customized treatment plans to optimize language after implant.

## Notes

Supplement 1. eMethods. eFigure. Correlation matrices of clinical variables across each center eTable. Performance comparison of four dimensionality-reduction techniques for feature extraction in machine learning models

Supplement 2. Data sharing statement
