# Supplementary material for: Forecasting Spoken Language Development in Children With Cochlear Implants Using Preimplant Magnetic Resonance Imaging
Source: JAMA Otolaryngol Head Neck Surg. 2025 Dec 26:e254694. Online ahead of print. doi: 10.1001/jamaoto.2025.4694 (PMC12743310; doi:10.1001/jamaoto.2025.4694)
Supplement: Supplement 1. — eMethods. eFigure. Correlation matrices of clinical variables across each center eTable. Performance comparison of four dimensionality-reduction techniques for feature extraction in machine learning models [file jamaotolaryngolheadnecksurg-e254694-s001.pdf]

Supplemental Online Content

Wang Y, Yuan D, Dettman S, et al. Forecasting spoken language development in children with cochlear implants using preimplant magnetic resonance imaging. *JAMA Otolaryngol Head Neck Surg*. Published online December 26, 2025. doi:10.1001/jamaoto.2025.4694

eMethods.

eFigure. Correlation matrices of clinical variables across each center

eTable. Performance comparison of four dimensionality-reduction techniques for feature extraction in machine learning models

This supplemental material has been provided by the authors to give readers additional information about their work.

© 2025 Wang Y et al. *JAMA Otolaryngology–Head & Neck Surgery*.

eMethods.

Spoken language measurements

Chicago data. The spoken language ability was assessed using the Speech Recognition Index-modified version (SRI-m) before implantation and at 6, 12, 18, 24, and 36 months after CI. SRI-m is a hierarchical battery of age-appropriate measures, which assesses the speech recognition abilities from parental reports for children with lower auditory abilities to direct measures of speech perception for children with higher auditory abilities. The SRI-m has been used in a Childhood Development after Cochlear Implantation (CDaCI) Study.[1,2] It consists of the Infant-Toddler Meaningful Auditory Integration Scale/Meaningful Auditory Integration Scale (IT-MAIS/ MAIS), Early Speech Perception Test (ESP), Multisyllabic Lexical Neighborhood Test/Lexical Neighborhood Test (M/LNT), the Phonetically Balanced Word Lists-Kindergarten (PBK), the Pediatric Az Bio in quiet (AzBio-q), and the Pediatric Az Bio in noise (AzBio-n). The clinician decided which test the children should be tested with based on their age, developmental ability, and hearing aptitudes. To reflect children's spoken language development on the same scale, the scores of these tests were rescaled resulting in a final score ranging from 0 to 600 with higher scores representing better spoken language abilities.

Melbourne data. The ability of receptive and expressive language was assessed using two norm-referenced instruments—Pre-school Language Scale [PLS-4, PLS-5] [4] and Peabody Picture Vocabulary Test [PPVT-Revised, 3rd and 4th Editions] [3] before implantation and at 12, 24, and 36 months after CI. The tools were chosen to be appropriate for the age and stage for each child. The PLS-4 and PLS-5 assesses children from birth to 6 years 11 months and birth to 7 years 11 months, respectively, through tasks administered by the clinician using toys and picture-based materials. Standard scores and age equivalents are derived for receptive, expressive, and total language. The PPVT measures receptive

© 2025 Wang Y et al. *JAMA Otolaryngology–Head & Neck Surgery*.

vocabulary for children aged 2 years 6 months through to adulthood. The child hears a stimulus word and selects the picture that best represents it from four options. Standard scores and age equivalents are derived for receptive vocabulary. The two assessment tools offer age-based standard scores with a mean of 100 and a standard deviation of 15.

Hong Kong data. The spoken language ability of the children was evaluated using the LittlEARS Auditory Questionnaire, which relies on caregivers' observation of children's auditory behaviors (including that of spoken language) in daily life.[5] This 35-item questionnaire, with 'yes' or 'no' responses, takes about 10 minutes for caregivers to complete. The LittlEARS has been validated in children with both normal hearing and hearing loss.[6,7] Specifically, the questionnaire has demonstrated evidence of validity in accounting for the substantial variation in spoken language development among children with CIs.[8,9] In this study, the children's spoken language scores as measured by LittlEARS were obtained before CI and at 6, 12, and 24 months after CI.

Statistical Analyses

To improve the applicability of the model, we provided more information at each center that consistently contributes to outcomes. Correlation analyses were conducted illustrating relationships between behavioral factors at each center.

© 2025 Wang Y et al. *JAMA Otolaryngology–Head & Neck Surgery*.

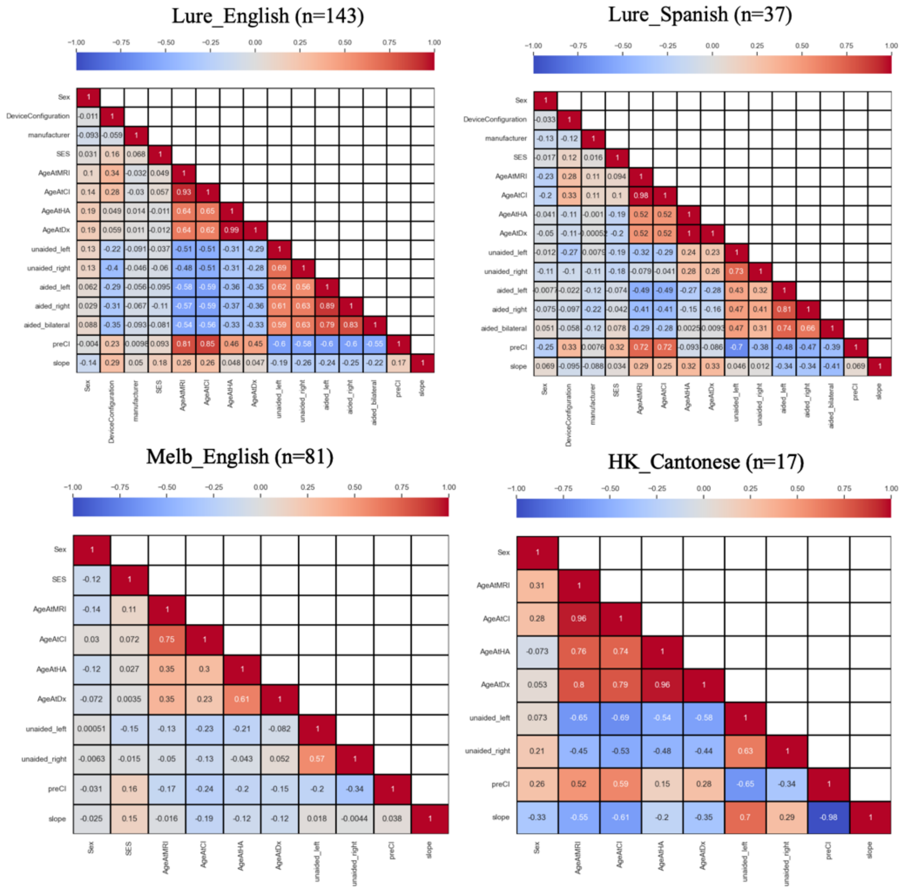

eFigure. Correlation matrices of clinical variables across each center. Abbreviations: SES, socioeconomic status; unaided_left/right, unaided pure tone average residual hearing in the left/right ear; preCI, baseline spoken language scores; slope, fitted slope of language scores across time points.

MRI acquisition

Chicago MRI data acquisition. The T1-weighted images were obtained on a 3T Siemens scanner (MAGNETOM Skyra, Vida) using a magnetization-prepared rapid gradient-echo (MPRAGE) sequence or on a 3T General Electric MR scanner (DISCOVERY MR750, SIGNA Architect) using a 3D brain volume (BRAVO) sequence or a FSPGR (Fast Spoiled Gradient Recalled Echo) sequence. The scanning parameters were optimized to obtain a good

© 2025 Wang Y et al. *JAMA Otolaryngology–Head & Neck Surgery*.

signal-to-noise ratio (BRAVO, N=43: TE = 2.72 ms~3.91 ms, TR = 7.40 ms~9.45 ms, flip angle = 12°, matrix = 512×512, number of slices = 148~512, slice thickness = 1 mm~2 mm, voxel size = 0.3 mm×0.3 mm×0.6 mm ~ 0.5 mm×0.5 mm×1.4 mm; FSPGR, N=1: TE = 4.3 ms, TR = 10.63 ms, flip angle = 20°, matrix = 256×256, number of slices = 101, slice thickness = 1.4 mm, voxel size = 0.9 mm×0.9 mm×1.4 mm; MPRAGE, N=136: TE = 2.38 ms~3.54 ms, TR = 1490 ms~2200 ms, flip angle = 8~9°, matrix = 192×192 ~ 512×512, number of slices = 108~224, slice thickness = 0.8 mm~1 mm, voxel size = 0.8 mm×0.8 mm ×0.8 mm ~ 1 mm×1 mm×1 mm).

Melbourne MRI data acquisition. The T1-weighted images were obtained on a 1.5T Siemens scanner (MAGNETOM Area, Avanto, and SymphonyTim) and 3T Siemens scanner (MAGNETOM Trio and Verio) using a magnetization-prepared rapid gradient-echo (MPRAGE) sequence. The scanning parameters were optimized to obtain a good signal-to-noise ratio (MPRAGE, N=81: TE = 2.31 ms~4.92 ms, TR = 11 ms~2100 ms, flip angle = 9°~20°, matrix = 576×426~224×198, number of slices = 142~452, slice thickness = 0.38 mm ~ 0.9 mm, voxel size = 0.4 mm×0.4 mm×0.8 mm ~ 0.9 mm×0.9 mm×0.9 mm).

Hong Kong MRI data acquisition. The T1-weighted images were acquired on a 3T Siemens Prisma scanner using a magnetization-prepared rapid gradient-echo (MPRAGE) sequence, or on a 3T General Electric MR scanner using 3D brain volume (BRAVO) sequence, or on a 3T Philips Achieva scanner using a turbo field echo (TFE) sequence. The scanning parameters were optimized to obtain a good signal-to-noise ratio (Siemens Prisma scanner: TE = 2.35 ms~2.59 ms, TR = 1800 ms, flip angle = 8°, matrix = 256×208 ~ 640×640, 192~320 slices of 0.69 mm ~ 3 mm thickness; General Electric MR scanner: TE = 2.68 ms~2.81 ms, TR = 7.62 ms~7.71 ms, flip angle = 12°, matrix = 512×512, 146~352 slices of 1 mm~1.1 mm thickness; Philips Achieva scanner: TE = 3.41 ms~3.59 ms, TR =

© 2025 Wang Y et al. *JAMA Otolaryngology–Head & Neck Surgery*.

7.46 ms~7.77ms, flip angle = 8°, matrix = 224×224 ~ 224×280, 224~250 slices of 1.1 mm thickness).

Voxel-based machine learning models

We compared the classification performance of slice-based deep learning with the following voxel-based machine learning classification algorithms on the English-learning Chicago dataset: (1) Linear regression trained with L1 regularization (Lasso), L2 regularization (Ridge), and L1L2 regularization (Elastic Net). (2) Support vector machine classifier (SVM). (3) Decision tree (DT). (4) Random forest classifier (RF). (5) K-nearest neighbor classifier (KNN). (6) Gradient tree boosting-based classifier implemented in XGBoost.

Each of these popular machine learning models was trained using the direct concatenation of the preprocessed MRI data as the input.[10,11] All the methods were trained with the same preprocessed data. The dataset was divided into 80% training and validation and 20% testing. To reduce the computational cost and enhance machine learning model performance, we applied four common dimensionality reduction methods to reduce the central brain slices voxels to low-dimensional representations including principal components analysis (PCA), Gaussian Random Projection (GRP), Recursive feature Elimination (RFE), and Univariate Feature Selection (UFS). Specifically, PCA identifies orthogonal axes of maximum variance in high-dimensional data through eigen decomposition of the covariance matrix[12,13]. By projecting data onto these principal components, it achieves optimal linear dimensionality reduction while preserving global data structure. GRP employs a random matrix with entries drawn from a Gaussian distribution to project high-dimensional data into a lower-dimensional subspace. This computationally efficient method preserves pairwise distances between data points (Johnson-Lindenstrauss lemma) while introducing controlled

© 2025 Wang Y et al. *JAMA Otolaryngology–Head & Neck Surgery*.

distortion[14]. RFE iteratively trains a model, ranks features by importance, and eliminates the least significant features until a predefined feature count is reached. This wrapper method progressively refines feature subsets while maintaining predictive power[15]. Finally, UFS selects features through individual statistical tests (e.g., mutual information), ranking each feature independently against the target variable. It retains only the highest-scoring features, offering model-agnostic efficiency at the cost of ignoring feature interactions.

Consequently, a random grid search and nested cross-validation strategy were employed to validate the machine learning models and find the optimal combination of parameters for each model. Specifically, each fold was used in turn as the test set while the four remaining folds were used as training set. A grid search was utilized with five-fold cross-validation and different parameter combinations.[17] All the models were evaluated using average accuracy and average error metrics. The results were compared to determine the best model and its optimal parameters.

Performance Evaluation Metrics

The model's performance in classification could be evaluated using the following performance metrics: the area under the receiver operating characteristic curve (AUC), accuracy (ACC), sensitivity, and specificity. AUC measures the model's ability to discriminate between classes across various thresholds and is calculated from the False Positive Rate (FPR) and True Positive Rate (TPR). ACC measures the proportion of correctly classified images, reflecting the overall effectiveness of the model. Sensitivity, or recall, assesses the classifier's ability to correctly identify cases with the disease. Specificity evaluates how well the classifier can identify cases without the disease.

$$ACC = (TP + TN) / (TP + TN + FP + FN)$$

© 2025 Wang Y et al. *JAMA Otolaryngology–Head & Neck Surgery*.

Sensitivity = TP / (TP + FN)

Specificity = TN / (FP + TN)

$$AUC = \int_{x=0}^{1} TPR\big(FPR^{-1}(x)\big)d_x = P(X_1 > X_0)$$

where TP is true positive values, TN is true negative values, FP is false positive values, and FN is false negative values; $X_1$is a positive instance and X_0 is a negative instance.

Sensitivity Analysis

To evaluate potential bias introduced by our slice selection strategy (i.e., slice counts or positions), we further performed extensive sensitivity analyses using alternative slice configurations. We compared model performance using three alternative 15-slice windows: superior windows from 35-50 slices encompassing the superior frontal and parietal lobes, central windows from 80-95 slices centered around the ventricular system, spanning temporal lobes, basal ganglia, and mid-ventricular region—areas consistently implicated in our targeted language-related regions, and inferior windows from 150-165 covering the brainstem, cerebellum, and inferior temporal lobes. All models were trained and evaluated using single neural network architecture, MobileNet. Results showed that the central window achieved the highest performance (AUC=0.92), outperforming both superior (AUC=0.52) and inferior (AUC=0.77). Moreover, we further evaluated the impact of slice counts using different numbers of central slices including 5, 15, and 25 slices. Results showed a slight decrease expanding to 25 slices with AUC of 0.89 and a notable drop (AUC=0.86, >6%) when reducing to 5 slices. This suggests that 15 central slices represent a reasonable trade-off between the coverage of relevant neuroanatomical structures and computational efficiency.

References

1. Eisenberg LS, Johnson KC, Martinez AS, et al. Speech recognition at 1-year follow-up in the childhood development after cochlear implantation study: methods and preliminary findings. *Audiology and Neurotology*. 2006;11(4):259-268.

2. Wang NY, Eisenberg LS, Johnson KC, et al. Tracking development of speech recognition: longitudinal data from hierarchical assessments in the Childhood Development after Cochlear Implantation Study. *Otology & neurotology: official publication of the American Otological Society, American Neurotology Society [and] European Academy of Otology and Neurotology*. 2008;29(2):240.

3. Zimmerman IL, Steiner VG, Pond RE. Preschool Language Scale, Fifth Edition. Published online November 12, 2012. doi:10.1037/t15141-000

4. Dunn LM, Dunn DM. Peabody picture vocabulary test (4th ed.). *Circle Pines: American Guidance Service*. Published online 2007.

5. Tsiakpini L, Weichbold V, Kuehn-Inacker H, Coninx F, D'Haese P, Almadin S. *LittlEARS Auditory Questionnaire.* Austria: MED-EL; 2004.

6. Bagatto MP, Brown CL, Moodie ST, Scollie SD. External validation of the LittlEARS® Auditory Questionnaire with English-speaking families of Canadian children with normal hearing. *International journal of pediatric otorhinolaryngology*. 2011;75(6):815-817. doi:10.1016/j.ijporl.2011.03.014

7. Liu H, Jin X, Zhou Y, LI J, Liu L, NI X. Assessment and Monitoring of the LittlEARS? Auditory Questionnaire Used for Young Hearing Aid Users in Auditory Speech Development. *Journal of Audiology and Speech Pathology*. Published online 2015:291-294.

8. May-Mederake B, Kuehn H, Vogel A, et al. Evaluation of auditory development in infants and toddlers who received cochlear implants under the age of 24 months with the LittlEARS® Auditory Questionnaire. *International journal of pediatric otorhinolaryngology*. 2010;74(10):1149-1155.

9. Obrycka A, Lorens A, García JLP, Piotrowska A, Skarzynski H. Validation of the LittlEARS Auditory Questionnaire in cochlear implanted infants and toddlers. *International Journal of Pediatric Otorhinolaryngology*. 2017;93:107-116.

10. Wen J, Thibeau-Sutre E, Diaz-Melo M, et al. Convolutional neural networks for classification of Alzheimer's disease: Overview and reproducible evaluation. *Medical image analysis*. 2020;63:101694.

11. Zhao Z, Chuah JH, Lai KW, et al. Conventional machine learning and deep learning in Alzheimer's disease diagnosis using neuroimaging: A review. *Frontiers in computational neuroscience*. 2023;17:1038636.

12. Feng G, Ingvalson EM, Grieco-Calub TM, et al. Neural preservation underlies speech improvement from auditory deprivation in young cochlear implant recipients. *PNAS*. 2018;115(5):E1022-E1031. doi:10.1073/pnas.1717603115

13. Tuckute G, Sathe A, Srikant S, et al. Driving and suppressing the human language network using large language models. *Nature Human Behaviour*. Published online 2024:1-18.

© 2025 Wang Y et al. *JAMA Otolaryngology–Head & Neck Surgery*.

© 2025 Wang Y et al. *JAMA Otolaryngology–Head & Neck Surgery*.

eTable. Performance comparison of four dimensionality-reduction techniques for feature extraction in Machine Learning Models

Feature extraction	Model	% (95% CI)			
		Accuracy	Sensitivity	Specificity	AUC (95% CI)
PCA	Lasso	55.71 (49.56-61.87)	58.57 (49.28-67.77)	52.86 (42.74-62.97)	0.557 (0.496-0.619)
	Ridge	56.07 (51.75-60.39)	42.86 (31.99-53.72)	69.29 (57.30-81.27)	0.561 (0.517-0.604)
	DT	49.64 (42.22-57.06)	37.14 (10.98-63.30)	62.14 (47.57-76.72)	0.496 (0.422-0.571)
	SVM	47.85 (26.68-69.03)	46.43 (24.93-67.93)	49.29 (27.70-70.87)	0.479 (0.267-0.690)
	KNN	56.79 (50.63-62.94)	48.57 (42.62-54.52)	65.00 (55.39-74.61)	0.568 (0.506-0.629)
	RF	49.29 (46.31-52.26)	43.57 (31.27-55.88)	55.00 (44.79-65.21)	0.493 (0.463-0.523)
	Xgboost	53.93 (46.68-61.18)	50.00 (31.45-68.55)	57.86 (48.24-67.47)	0.539 (0.467-0.612)
GRP	Lasso	55.72 (50.91-60.52)	53.57 (34.50-72.64)	57.86 (37.44-78.28)	0.557 (0.509-0.605)
	Ridge	56.79 (51.98-61.59)	52.14 (25.06-79.23)	61.43 (42.51-80.35)	0.568 (0.520-0.616)
	DT	53.21 (45.01-61.42)	47.86 (22.85-72.86)	58.57 (27.59-89.55)	0.532 (0.450-0.614)
	SVM	54.64 (49.79-59.50)	43.57 (20.23-66.91)	65.71 (48.37-83.06)	0.546 (0.498-0.595)
	KNN	53.93 (43.84-64.02)	50.71 (38.41-63.02)	57.14 (46.28-68.01)	0.539 (0.438-0.640)
	RF	51.79 (48.28-55.29)	43.57 (37.79-49.35)	60.00 (51.47-68.53)	0.518 (0.483-0.553)
	Xgboost	55.00 (49.48-60.52)	50.71 (39.67-61.76)	59.29 (56.85-61.17)	0.550 (0.495-0.605)
RFE	Lasso	56.79 (51.98-61.59)	57.86 (47.27-68.44)	55.71 (53.29-58.14)	0.568 (0.520-0.616)
	Ridge	55.72 (50.66-60.77)	56.43 (43.73-69.13)	55.00 (51.03-58.97)	0.557 (0.507-0.608)
	DT	49.29 (37.81-60.76)	38.57 (25.87-51.27)	60.00 (36.67-83.34)	0.493 (0.378-0.608)
	SVM	51.07 (48.54-53.60)	47.14 (42.29-52.00)	55.00 (48.27-61.73)	0.511 (0.485-0.536)
	KNN	54.64 (49.54-59.75)	49.29 (42.71-55.86)	60.00 (52.71-67.29)	0.546 (0.495-0.597)
	RF	48.93 (37.13-60.72)	46.64 (36.51-56.34)	51.43 (36.86-66.00)	0.489 (0.371-0.607)
	Xgboost	51.07 (43.96-58.19)	43.57 (25.18-61.96)	58.57 (51.15-65.99)	0.511 (0.440-0.585)
UFS	Lasso	58.57 (53.76-63.38)	51.43 (44.01-58.85)	65.71 (61.75-69.68)	0.586 (0.538-0.634)
	Ridge	62.14 (59.25-65.03)	55.72 (47.07-64.36)	68.57 (61.28-75.59)	0.621 (0.593-0.650)
	DT	60.71 (56.57-64.86)	42.86 (31.99-53.72)	78.57 (64.20-92.94)	0.607 (0.566-0.649)
	SVM	60.36 (57.47-63.25)	55.71 (53.29-58.14)	65.00 (59.22-70.78)	0.604 (0.575-0.632)
	KNN	59.64 (54.30-64.98)	56.43 (45.84-67.02)	62.86 (54.80-70.91)	0.596 (0.543-0.650)
	RF	59.64 (54.30-64.98)	47.86 (38.66-57.05)	71.43 (68.29-74.56)	0.596 (0.543-0.650)
	Xgboost	59.64 (49.65-69.63)	55.00 (42.22-67.78)	64.29 (51.36-77.21)	0.596 (0.497-0.696)

© 2025 Wang Y et al. *JAMA Otolaryngology–Head & Neck Surgery*.

© 2025 Wang Y et al. *JAMA Otolaryngology–Head & Neck Surgery*.
