# Supplementary material for: Forecasting Spoken Language Development in Children With Cochlear Implants Using Preimplant Magnetic Resonance Imaging
Source: JAMA Otolaryngol Head Neck Surg. 2025 Dec 26:e254694. Online ahead of print. doi: 10.1001/jamaoto.2025.4694 (PMC12743310; doi:10.1001/jamaoto.2025.4694)
Supplement: Supplement 2. — Data sharing statement [file jamaotolaryngolheadnecksurg-e254694-s002.pdf]

Data Sharing Statement

Wang. Forecasting Spoken Language Development in Children With Cochlear Implants Using Preimplant Magnetic Resonance Imaging. *JAMA Otolaryngol Head Neck Surg*. Published December 26, 2025. doi:10.1001/jamaoto.2025.4694

Data
Data available: No

Additional Information
Explanation for why data not available: The datasets in the current study are not publicly available due to strict privacy regulations set forth by the Institutional Review Board.
